# Neurocognitive mechanisms underlying working memory encoding and retrieval in Attention-Deficit/Hyperactivity Disorder

**DOI:** 10.1038/s41598-020-64678-x

**Published:** 2020-05-08

**Authors:** Rodrigo Ortega, Vladimir López, Ximena Carrasco, María Josefina Escobar, Adolfo M. García, Mario A. Parra, Francisco Aboitiz

**Affiliations:** 1 Departamento de Psicología, Facultad de Ciencias Sociales, Universidad de Chile, Santiago, Chile; 2 Center for Social and Cognitive Neuroscience (CSCN), Escuela de Psicología, Universidad Adolfo Ibáñez, Santiago, Chile; 3 Escuela de Psicología, Facultad de Ciencias Sociales, Pontificia Universidad Católica de Chile, Santiago, Chile; 4 Laboratorio de Neurociencias Cognitivas, Departamento de Psiquiatría, Centro Interdisciplinario de Neurociencias, Facultad de Medicina, Pontificia Universidad Católica de Chile, Santiago, Chile; 5 Servicio de Neurología y Psiquiatría, Hospital de Niños Dr. Luis Calvo Mackenna, Facultad de Medicina, Universidad de Chile, Santiago, Chile; 6 Universidad de San Andrés, Buenos Aires, Argentina; 7 National Scientific and Technical Research Council (CONICET), Buenos Aires, Argentina; 8 Faculty of Education, National University of Cuyo, Mendoza, Argentina; 9 Departamento de Lingüística y Literatura, Facultad de Humanidades, Universidad de Santiago de Chile, Santiago, Chile; 10 School of Psychological Sciences and Health, University of Strathclyde, Glasgow, UK; 11 Facultad de psicología, Universidad Autónoma del Caribe, Barranquilla, Colombia

**Keywords:** Attention, Cognitive control

## Abstract

Working memory (WM) impairments in ADHD have been consistently reported along with deficits in attentional control. Yet, it is not clear which specific WM processes are affected in this condition. A deficient coupling between attention and WM has been reported. Nevertheless, most studies focus on the capacity to retain information rather than on the attention-dependent stages of encoding and retrieval. The current study uses a visual short-term memory binding task, measuring both behavioral and electrophysiological responses to characterize WM encoding, binding and retrieval comparing ADHD and non-ADHD matched adolescents. ADHD exhibited poorer accuracy and larger reaction times than non-ADHD on all conditions but especially when a change across encoding and test displays occurred. Binding manipulation affected equally both groups. Encoding P3 was larger in the non-ADHD group. Retrieval P3 discriminated change only in the non-ADHD group. Binding-dependent ERP modulations did not reveal group differences. Encoding and retrieval P3 were significantly correlated only in non-ADHD. These results suggest that while binding processes seem to be intact in ADHD, attention-related encoding and retrieval processes are compromised, resulting in a failure in the prioritization of relevant information. This new evidence can also inform recent theories of binding in visual WM.

## Introduction

Attention-Deficit/Hyperactivity Disorder (ADHD) is a highly prevalent neurodevelopmental disorder characterized by attentional difficulties, hyperactivity and impulsivity^1–3^. Nevertheless, attention is neither the only cognitive process affected in this condition nor the most affected one. For instance, the search for specific impairments in selective attention and orienting attention in ADHD has not yielded consistent results^4–6^. Among other cognitive processes, frontal executive functions impairments are consistently reported in ADHD^7,8^. Moreover, Working Memory (WM) impairment is considered a significant cognitive feature differentiating between ADHD and non-ADHD children^9^.

The predominant theoretical model of WM is Baddeley’s multi-component model^10,11^. WM is defined as a limited-capacity system responsible for encoding, retaining or maintaining, and manipulating cognitive representations of stimuli. Such memory system encompasses independent phonological (PH) and visuospatial (VS) subsystems, and a central executive (CE) component, responsible for the attentional control. A fourth component, the episodic buffer, was later added^12^. Other authors, like Cowan^13^ or Engle^14^, have proposed WM models that emphasize the predominant role of attention throughout WM stages. Attentional involvement in each memory stage has also been a matter of debate. It has been suggested that attention is necessary for encoding, updating and retrieval but has a limited role during retention^15^.

In the experimental psychology literature, several studies have been reported which aimed to investigate the extent to which binding surface features in visual WM is an automatic or an attentional demanding function^16–19^. The evidence gathered to date suggests that binding of features in visual WM requires no more attentional resources than processing individual features. The consistency of these findings across a thorough experimental series, led Allan Baddeley to revise the WM model^20^. Such a revision tried to address the concept and function of the episodic buffer; a WM component wherein binding functions were thought to be carried out via the support from attention. Despite the attractiveness of these studies, they focused on healthy samples of university students who, although subjected to experimental manipulations of attention, may have enough available resources to cope with attentional interference and still perform the task at a high level of accuracy. It would be highly desirable to further investigate visual WM binding in individuals with attentional disorders, such as those diagnosed with ADHD.

Traditionally, WM is considered part of the executive functions. Both WM and executive functions, have been criticized due to their limited specification^3,9^. Nevertheless, processes like conflict detection, detecting mismatch from expectations, shifting or interrupting a response, and the effortful allocation and maintenance of attention and working memory resources towards the attainment of a future goal appear to be compromised in ADHD^9^. Metanalytic studies suggest that processes such as executive attention, working memory, along with decision making factors like motivation and reward are central to understand the ADHD cognitive profile^21^. Reaction times variability is also considered part of this cognitive profile^3^.

When children, adolescents and adults with ADHD are assessed, WM and other executive dysfunctions stand out as the ones with the most reliable discriminative power^21^. Regarding the specific WM deficit in ADHD both, phonological and visuospatial components seem to be affected, being the task demands on the central executive (CE) one of the key moderators to explain the results^22,23^. That is, the most sensitive WM tasks in ADHD are those with high demands of CE component. For example, those that require the participants to remember stimuli and later recall them in a different pattern than the originally presented, or those that require to compare a newly presented stimulus with a representation in WM and to update that representation. ADHD subtype (predominantly Inattentive or Combined) seems to have no significant effect on WM dysfunction, perhaps due to their shared inattention symptomatology^24,25^.

The close relationship between selective attention and WM has long been considered a natural candidate to explain WM impairments in ADHD^26^. These impairments have been related to academic underachievement due to poor acquisition of cognitive skills in children, which may also have a long-term impact in social development and quality of life^27,28^. Unfortunately, despite the consensus about the relevance of WM deficits in ADHD, the precise mechanisms that affect ADHD performance in WM tasks are poorly understood^29,30^. Moreover, WM training seems to have a limited beneficial impact on ADHD, even when a significant improvement in WM performance is achieved^31^. This emphasizes the need to understand and empirically document the nature of the WM impairment in ADHD in relation with other process such as attention, to improve the development of diagnostic or intervention tools.

Electrophysiological measures such as event related potentials (ERP) are especially useful to study WM, as they allow to differentiate stages of encoding, retention and retrieval which cannot be directly inferred from behavioral responses. Encoding and retrieval are systematically associated with P3-like ERP components^32,33^. Interestingly, reduced P3 amplitude in ADHD has been described both in children^34^ and adults^35^. The retention stage is commonly studied by means of contralateral delayed activity (CDA) which is sensitive to WM load and capacity^36^. This ERP component is a negative slow deflection usually detected at contralateral parietal sites that exhibit larger amplitude (compared to the ipsilateral sites) as the number of items in WM increases^37^.

Change detection tasks (CDT) have proved to be a successful paradigm to specifically explore attention and WM^16–19,38^. This task usually consists of the presentation of an array of stimuli for a short period of time (S1), which must be kept in memory (retention period) until the presentation of a test stimuli array (S2), where the subject must respond whether the test stimuli is the same or different (Trial Type). This design allows the evaluation of the three stages described for WM: encoding, retention and retrieval. In a recent study, Spronk *et al*.^29^ used a CDT to evaluate the impact of distractors on the retention capacity of WM, by comparing adolescents and adults with ADHD and healthy controls. They found that adolescents were more affected than adults by the presence of distractors but found no differences regarding encoding and retention between groups. However, this study evaluated only up to the retention period and not later stages where the subject must contrast the target with the memory representation and generate a response. Post-retention is highly overlooked in most WM and ADHD studies^34,39^. Nevertheless, ADHD difficulties in working memory updating and retrieval have been previously reported using a different WM task^39^. Additionally, previous results of our groups suggest that the use of cognitive resources and particularly attentional resources in ADHD reflects a differential style more than a deficit pattern or a deficient capacity^1,2^. Such types of functional impairments could well affect the use of WM in the post-retention period more than the process of forming representations in WM. Thus, an accurate characterization of encoding and retrieval working memory stages in ADHD could be relevant to better understand WM role in this condition.

In summary, consensus exists regarding a poor performance of subjects with ADHD in tasks that explore WM functioning. Nonetheless, there is no clear evidence concerning which process or mechanism is actually compromised. Moreover, whether this deficit depends on encoding, retention, or retrieval processes (or some combination of them) is still unknown. Likewise, the retrieval of the information from WM is also an important and mostly overlooked stage that should be explored in ADHD.

Here we develop a novel approach aimed to explore functional indicators (behavioral and electrophysiological) of the different WM stages to further our knowledge on their role in the ADHD related WM impairment. Parra *et al*.^40^ designed a visual short-term memory binding task that allows studying binding and change detection controlling the potential influence of the spatial location and spatial relations within the stimuli arrays. It has allowed to describe specific patterns of WM impairment in other conditions (e.g. Alzheimer’s Disease)^20,40,41^. In the present study we use a modified version of this experimental design that differs from previous studies controlling two possible confounding aspects. First, it minimizes the possibility of linguistic rehearsal by using no nameable polygons and non-primary colors as stimuli. Second, it allows controlling the use of spatial cues by changing the spatial location of stimuli between S1 and S2 displays. WM binding is studied by contrasting blocks in which all shapes are presented in black, so only the shape should be retained (Shape-Only) and others in which the binding of shape and color is necessary to solve the task (Color-Shape binding).

Taking into account the ADHD performance on other related tasks, as well as evidence gleaned from the experimental psychology literature, we expect in this study that impaired attention would impact on both encoding and retrieval, resulting in a poorer overall performance in ADHD participants^42,43^. This would be especially manifest when S2 stimuli are different from S1. The need for WM binding (color and shape) could impact overall behavioral performance in all participants, but we expect no specific differences regarding ADHD diagnosis. At the electrophysiological level, we predict that encoding and retrieval impairment in ADHD will be associated with a reduced amplitude of the corresponding P3 ERP components. Additionally, we expect that these electrophysiological markers should be correlated (i.e., P3 at encoding with P3 at retrieval stages). In the retrieval stage, group differences should be more evident when S1 and S2 are different. Notwithstanding, these electrophysiological patterns should hold regardless of whether individual or bound features are the memoranda.

## Methods

### Participants

A group of 18 adolescents diagnosed by a certified pediatric neurologist with ADHD Combined Subtype according to the DSM-V criteria, that were being treated at the neurology service of the Luis Calvo-Mackenna Children’s Hospital in Santiago and an equal number of non-ADHD adolescents, from public schools of the same metropolitan area, voluntarily participated in the study. Their ages were from 12 to 14 years (12.61 ± 0.80). They were matched by age (ADHD: 12.66 ± 0.76, non-ADHD: 12.55 ± 0.85, F(_1, 34_) = 0.16832, p = 0.68419), IQ (ADHD: 99.66 ± 7.12, non-ADHD: 103.66 ± 7.17, F(_1, 34_) = 2.8138, p = 0.10263) and educational level (school grade). A complete clinical neurological and psychological evaluation was conducted in all the participants to rule out any potential confound. That included Conner ‘s Rating scale for parents and teachers, MINI-KID, STAI anxiety inventory and WISC III test. Subjects with antecedents of any other Neurological or Psychiatric disease were excluded from the study. Comorbid symptoms of anxiety and conduct disorder were observed, but no ADHD participant met the criteria for any mayor comorbid disorder. They were being treated with methylphenidate for at least four months, but suspended medication 24 hours prior to the study.

The required sample size was calculated a priori using G*Power 3^44^ according to the sample sizes, statistical power and effect sizes described in previous studies using the same task and/or comparing the same dependent variables^45,46^. Expecting a small to moderate effect sizes, the required sample size was of 16 participants per group.

Recruitment of participants were conducted according to the standards set forth in the Declaration of Helsinki. After a clear explanation of the purpose and nature of the research they were asked to formally express their williness to participate. Informed consent was obtained from a parent and/or legal guardian and participants also signed an informed assent form. They were explicitly informed that they were free to finish their participation at any moment without any question. The whole protocol was examined, approved and followed by the ethical committee of the Pontifical Catholic University of Chile.

### Experimental design

This experimental design is an adaptation from previous studies of our group^20,40,41,45,47,48^, in particular one adapted for EEG recordings and presenting additional control of potential confounding such as linguistic rehearsal and spatial information^49^.

#### Stimuli

**Figure 1:**
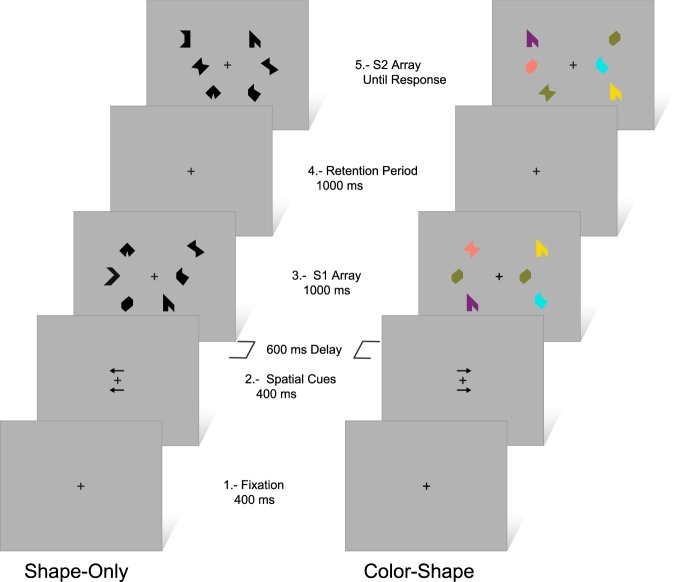
Schematic view of the experimental task. Left: Shape-Only condition, Different trial type. Right: Color-Shape condition, Same trial type. Time in milliseconds (ms).

No nameable geometric shapes and non-primary colors were used to minimize verbal rehearsal^40^. Two arrays, of three items each, were presented to the left and to the right of a fixation cross. Each array was presented using a virtual 3×3 grid (4° horizontally x 8° vertically), 3° to the left and right of a central fixation cross on a grey background. Each item size was 1° and was, at least, 2° apart from any other item. Items for the study display (S1) were randomly selected from a set of eight polygons and eight colors and randomly allocated to the 9 positions within the virtual grid. During the test phase (S2), the same three locations used during the study phase were used but items locations were interchanged. Hence, items were never presented in the same locations across the study and test display. By this way, spatial location was render uninformative (see Fig. 1).

#### Design

The task consisted of four blocks counterbalanced across participants. Two of them were of the shape-only condition where the stimuli consisted of three shapes in black color. The other two were Shape-Color Binding condition and stimuli were three colored shapes. For each block there was a short practice session (8 trials per block) followed by the test. Each block presented 80 trials (40 right and 40 left of which 20 are same trials – “Same”, S1 = S2 - and 20 are different – “Different”, S1 ≠ S2 – trials). In the last case, two shapes or two colors were replaced by different ones. There was a total of 320 trials. The total duration of this task was approximately 35 minutes.

During each trial a fixation cross was presented, and participants were asked to keep their eyes on it and to press a key to initiate the trial. Fixation remained on screen throughout the trial. After 400 ms, two arrows were presented for other 400 ms above and below fixation. Arrows direction indicated which side should be attended. After a delay of 600 ms the S1 array was presented for 1000 ms, followed by a 1000 ms retention interval. Then, the S2 array is presented and remained on until the participant responded “Same” or “Different”, by pressing a specific key with their dominant hand.

#### Experimental procedure

EEG recording sessions began by asking participants to sit comfortably in a dimly lit, electrically and acoustically shielded room. A shin rest device was used to reduce unwanted head movements. Participants receive standard verbal instructions about the experimental procedure and the experimental task. All participants were evaluated individually.

### Data acquisition

Electrophysiological signals were recorded using a NeuroScan 40-channel Digital Electroencephalograph with a high-resolution NuAmp amplifier. A 40-channel cap (Quick-Cap) from the same company was used for electrode placement following the international 10/20 electrode sites and linked mastoids as the reference. Impedances were kept below 5 kΩ throughout the recordings. A/D sampling frequency was set at 1000 Hz. A band-pass digital filter between 0.1 and 30 Hz was later applied to remove unwanted frequency components. Two additional bipolar derivations were used to monitor vertical and horizontal ocular movements (VEOG, HEOG).

### Data analyses

For behavioral data, the percentage of correct responses (accuracy) and reaction times (RTs) were measured in all subjects and conditions. Regarding ERPs, offline EEG signals were analyzed using EEGLAB/ERPLAB Matlab toolbox^50,51^. Eye movements or blink artefacts were corrected using ICA (Independent component analysis). Remaining trials that contained voltage fluctuations exceeding ±100 μV (microvolts), transients exceeding ±100 μV, or electro-oculogram activity exceeding ±50 μV were rejected. Artifact free waveforms were segmented into 1200 ms epochs starting 200 ms before the onset of S1 and S2 arrays. Separate average waveforms for each condition were generated.

We used a mixed model ANOVA with repeated measures for behavioral (RT, accuracy) and ERP variables. The Encoding period of ERP components analysis has two levels: (1) Group (ADHD vs no-ADHD); and (2) Condition (Single Shape vs. Shape-Color Binding). For the retrieval period (ERP components and behavioral results), a three levels analysis was performed: 1) Group (ADHD vs no-ADHD); 2) Condition (Single Shape vs. Shape-Color Binding); and 3) Trail type (Same vs. Different). All statistical calculations on ERPs were performed using individual waveforms. Mean amplitude in the windows 100–130 ms for P1, 180–210 ms for N1, 320–430 ms for early-P3, 430–600 ms for encoding late-P3, and 320–430 for Retrieval P3 were selected. P1 and N1 amplitudes were measured on the occipital region (electrodes O1, Oz, O2) yielding similar results. Encoding early and late P3 and retrieval P3 were measured in Parieto-Occipital midline region (electrodes CPz, Pz, Oz). Selection of electrodes sites and ERP measures was conducted following the recommendations previously described for this type of procedures^52^. For simplicity, only the results from posterior midline (Pz and Oz) were shown. Post hoc comparisons were assessed with Tukey HSD test. Greenhouse-Geisser and Bonferroni corrections were applied to compensate for violations of sphericity and multiple comparisons. Only statistically significant results of ERPs (p < 0.05) were used to test the association among sequential ERP components. Correlations were explored using Pearson’s correlation coefficient (r). Fisher’s R to Z procedure was later used to compare correlations coefficients^53^. Follow up Bayesian analysis were performed to explore non-significant interactions in early and late encoding P3 components using JASP software 0.11.1^54^.

## Results

### Behavioral Results

**Figure 2:**
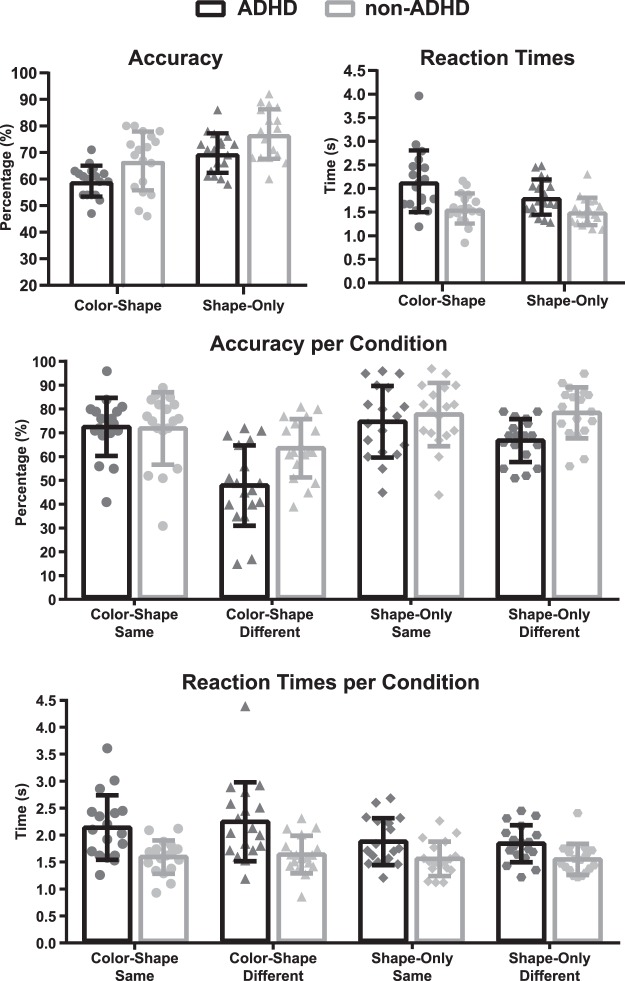
Behavioral results, Accuracy and Reactions Times. ADHD: black bars, non-ADHD: grey bars. Time in seconds (s).

Regarding accuracy, the ADHD group showed a poorer performance on all conditions (F_(1, 34)_ = 10.047, p = 0.00322, η^2^ = 0.23) compared to the non-ADHD group. There was a significant and expected main effect for condition type, whereby Only-Shape resulted in better performance (F_(1, 34)_ = 39.803, p = 0.00000, η^2^ = 0.54) than Color-Shape condition, but no Group x Condition interaction (F_(1, 34)_ = 0.020, p = 0.88969, η^2^ = 0.00) was found. Trial Type (same or different) was also significant. When S2 was different from the S1, a significant reduction in the hit rate (F_(1,34)_ = 13.817, p = 0.00072, η^2^ = 0.29) was observed. A significant interaction was found between Group and Trail Type (F_(1,34)_ = 5.2559, p = 0.02818, η^2^ = 0.13). Post-hoc analyses showed a significant drop of performance in the ADHD group during different trials (i.e., S2 different from S1) (MSE = 0.01165, df = 66.789, p = 0.0017) (see Fig. 2).

Regarding reaction times (RTs), the ADHD group showed slower responses than the non-ADHD group in all conditions (F_(1,34)_ = 13.035, p = 0.00097, η^2^ = 0.28). The Color-shape condition showed slower RTs than the Only-Shape condition (F_(1,34)_ = 6.2918, p = 0.01706, η^2^ = 0.16). No other effects were observed.

In summary, ADHD had longer RTs and a poorer performance on all conditions but especially in the different trials. Binding manipulation affected equally ADHD and non-ADHD participants.

### Electrophysiological Results

**Figure 3:**
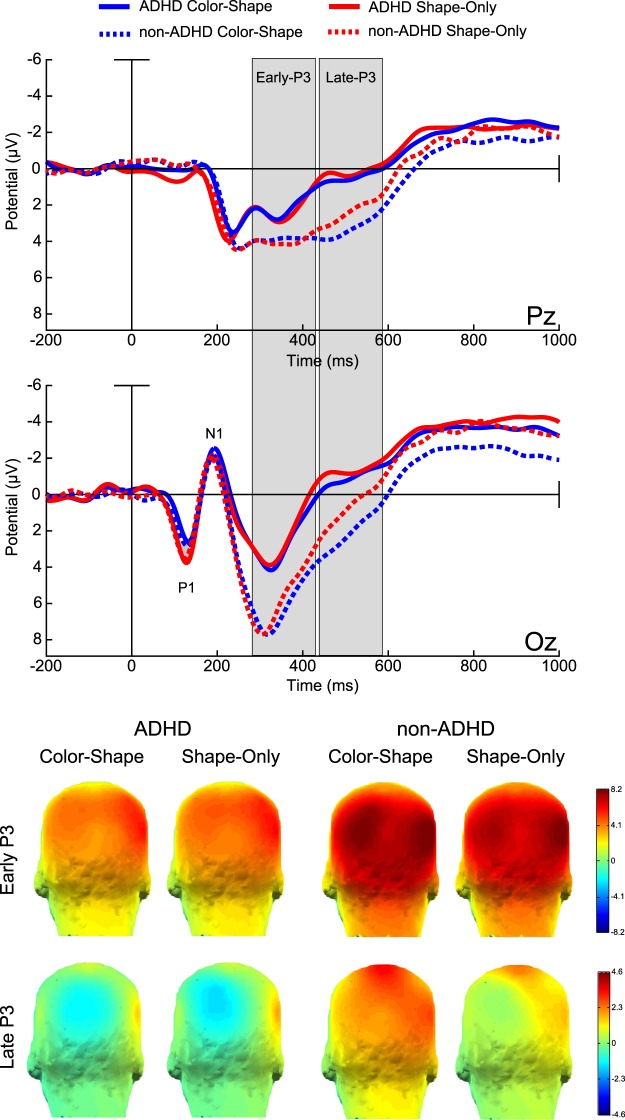
Encoding stage ERPs and topographic maps. Color-Shape: blue lines, Shape-Only: red lines. ADHD: solid lines, non-ADHD: dashed lines. Amplitudes in Microvolts (µV). Time in milliseconds (ms).

The P1 component at encoding showed larger amplitudes for the Shape-Only condition (F_(1,34)_=12.304, p = 0.00129, η^2^ = 0.27) compared to the Color-Shape condition. No differences in amplitude between groups were observed (F_(1,34)_=00440, p = 0.94751, η^2^ = 0.00). The following N1 component showed no amplitudes differences between groups (F_(1,34)_=0.08056, p = 0.77826, η^2^ = 0.00), or conditions (F_(1,34)_=1.3163, p = 0.25927, η^2^ = 0.03). After this negativity, a wide P3-like positivity was identified in the parieto-occipital region of the scalp, with a peak around 340 ms and extended in time up to 600 ms. The earlier segment of this component, between 320 to 430 ms, exhibited larger amplitude in the non-ADHD group than in the ADHD (F_(1,34)_=5.3294, p = 0.02718, η^2^ = 0.14), no significant amplitude difference by condition (F_(1,34)_=2.9067, p = 0.0973, η^2^ = 0.07) or Group x Condition interaction (F_(1,34)_=0.1811, p = 0.67310, η^2^ = 0.00) were found. The later part of this positivity (430–600) also showed larger amplitude in the non-ADHD (F_(1,34)_=6.4352, p = 0.01595, η^2^ = 0.16), and larger amplitudes for the Color-shape condition (F_(1,34)_=5.8871, p = 0.02071, η^2^ = 0.15). Again, no significant Group x Condition interaction (F_(1,34)_=2.4440, p = 0.12723, η^2^ = 0.06) was found (see Fig. 3). A follow up analysis using a Bayesian approach to explore the odds in favor of null hypothesis regarding the Group x Condition Interactions showed moderate support for the null in the P3 early window (BF_10_ = 0.3111) and strong support in the P3 late windows (BF_10_ = 0.014).

**Figure 4:**
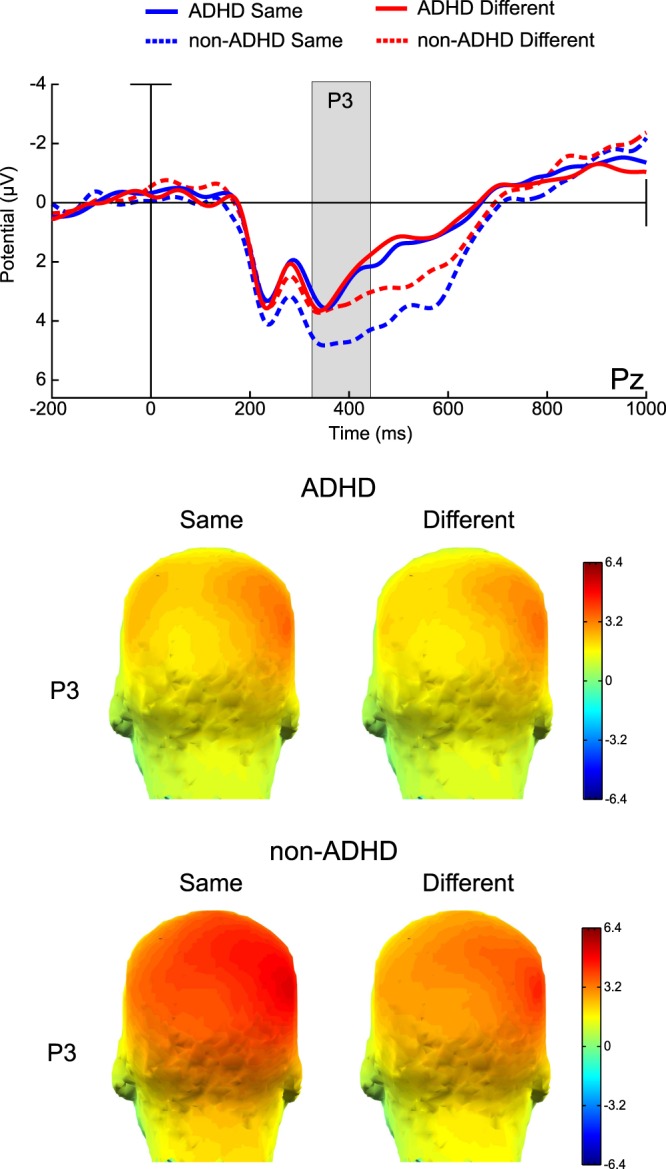
Retrieval stage ERPs and topographic maps. Same: blue lines, Different: red lines. ADHD: solid lines, non-ADHD: dashed lines. Amplitudes in Microvolts (µV). Time in milliseconds (ms).

A similar P3 like positive wave was evoked by S2. This retrieval P3 showed no significant main effect for groups (F_(1,34)_=0.26277, p = 0.61154, η^2^ = 0.00). There was a significant main effect of trial type, due to larger P3 amplitude in the Same trials compare to the Different ones (F_(1,34)_=4.9394, p = 0.03301, η^2^ = 0.13). A statistically significant interaction between group and Trial type was observed (F_(1,34)_=4.3989, p = 0.04347, η^2^ = 0.11). Follow up post-hoc contrasts showed that while P3 amplitude differentiated between Same and Different trials in the non-ADHD group (MSE = 12.724, df = 37.905, p = 0.0216), it was not the case for the ADHD group (MSE = 12.724, df = 37.905, p = 0.9996) (see Fig. 4).

In summary, P3 at encoding was larger in the non-ADHD group. P3 at retrieval discriminated the presence or absence of a change only in the non-ADHD group. Binding-dependent ERP modulations were not sensitive to group membership.

### Associations among sequential ERPs at different stages

**Figure 5:**
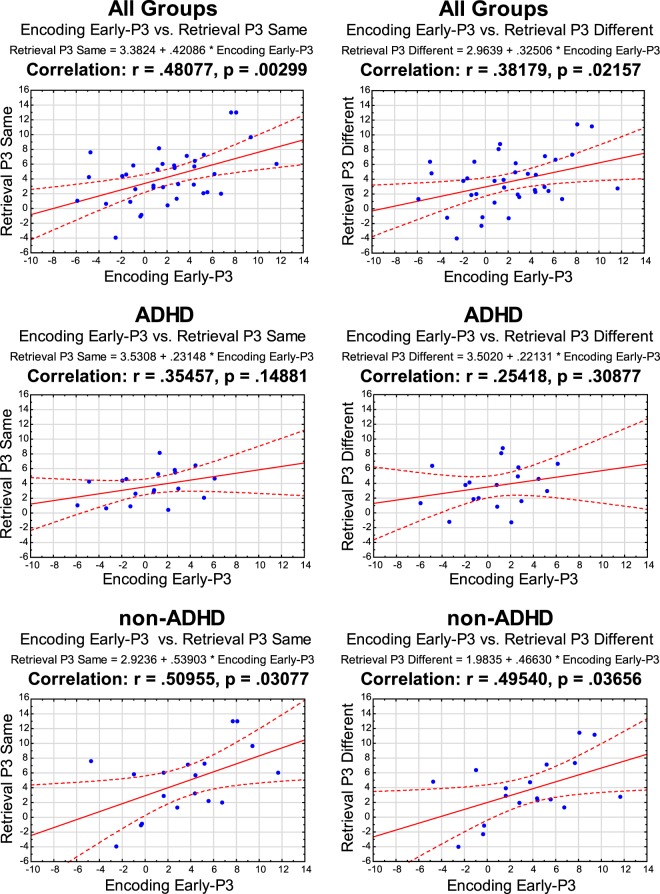
Encoding and Retrieval P3 component Amplitudes correlations. Pearson’s correlation coefficient (r).

The amplitude of the encoding early-P3 significantly correlated with that of the retrieval period, both in the Same (r = 0.48077, p = 0.00299) and Different (r = 0.38179, p = 0.02157) trial types. Follow up analysis showed that these significant correlations were driven by the results from the non-ADHD group: Same (r = 0.50955, p = 0.03077) and Different (r = 0.49540, p = 0.03656). The equivalent analysis in the ADHD group showed no significant correlation: Same (r = 0.35457, p = 0.14881) and Different (r = 0.25418, p = 0.30877) (see Fig. 5). Comparing the correlation coefficients between the groups using Fischer’s R to Z approach resulted in non-discriminative observed Z: Same Z_(obs)_= 0.5230, Different Z_(obs)_= 0.7750.

## Discussion

In the present study, we found a poorer overall performance and larger RTs in ADHD versus non-ADHD participants. Particularly, ADHD participants produced significantly fewer hits (i.e., correctly detect if S1 and S2 were different). The electrophysiological results evidenced significant differences between the groups in ERP components elicited during encoding and significant interaction Group x Trial Type during retrieval. The need to bind color and shape resulted in no significant Group x Condition interaction, suggesting that ADHD has no differential impact on binding functions carried out in WM. There was a significant correlation between the amplitude of the P3 component elicited during encoding and that elicited during retrieval that was significant only in the non-ADHD group. These results have important implications for our understanding of the involvement of WM in ADHD and the functional organization of this cognitive function. We discuss these implications below.

### Implications for WM functions in ADHD

The behavioral results of the current study supported our original hypothesis. All participants showed better accuracy in the “Shape-Only” than in the “Color-Shape” condition. This result has been previously observed in other studies using similar experimental designs^20,45^. They are interpreted as the cost of integrating features into objects to be kept in WM and are in line with the predictions from the feature integration theory^55^. Additionally, all participants performed better when the study (S1) and the test arrays (S2) were composed of the same items relative to trials where they had to detect and report changes happening in the test array. That is, when they had to update the WM representation to account for a change. These results are in line with previous studies using similar WM tasks^40,56^. Our hypothesis of ADHD’s poorer performance in all conditions was also confirmed, supporting previous reports in the literature^9,21,42^. Interestingly, this was significantly increased when a WM updating was needed.

Traditionally, poor behavioral performance of ADHD individuals on WM tasks has been explained in terms of a dysfunctional attentional process that impairs proper use of WM resources^57^. For instance, a deficient filtering of the incoming information could overload WM, rendering it also deficient^58,59^. This idea implies that attention and WM resources operate in tandem to process the available stimuli with the former supporting the latter. Nevertheless, the characterization of attention impairments in ADHD does not support this notion. The idea of a deficient filtering in ADHD causing an overload of working memory and resources depletion has been disputed^58,59^. Previous studies from our group^1,2^ point in a different direction. First, although ADHD do have problems when dealing with distractors it is not necessarily due to a deficient attentional filtering. Instead, they seem to follow task relative relevance to select and pay attention to objects^2^. Furthermore, several studies have proven that specific attention deficits in ADHD could be elusive^5^. The most consistent finding points to a dysfunction in executive attention, as part of a more general executive functions impairment that also include WM^60^ (but see also^3^). In this way, administering attention and WM resources seems to be the most typical problem. Therefore, a clear description of how the different WM sub-processes (encoding, binding-retention and retrieval) operate in this population and how they relate to each other (and to attention) seems critical to understand WM deficits in ADHD.

As previously stated, behavioral responses do not allow to discriminate between the different WM stages and their potential contribution to the impairment. ERPs have a high temporal resolution and different components have been described as functional indicators of distinct attention and WM processes. Attention allocation impacts the amplitude of early components of the visual ERP (P1, N1), increasing their amplitude^61^. In the present study, we found significant amplitude differences between conditions but no differences between groups. These findings also point *against* a deficient early visual filtering as a mechanism that could explain attention-WM impairment in ADHD^1,2^. On the contrary, the P3 component has been linked to working memory and attention since its earliest descriptions^62^. P3 amplitude has been suggested to indicate working memory updating^32^ but also resource allocation^63^. The amplitude of P3 is known to be affected by attention allocation and, interestingly, a reduced P3 amplitude has been reported in ADHD patients through a wide variety of cognitive tests^34^.

In the present study, the encoding and the retrieval periods were characterized by the presence of the P3 like component elicited by the study array and the test array respectively. In both cases these components had larger amplitude in non-ADHD than in ADHD. These WM-related P3 components have been previously reported in several WM tasks^33,64^. Its amplitude has been related with the efficacy of encoding and retrieval^65,66^. For example, Friedman and Johnson^67^ found that items subsequently recognized or remembered elicited larger encoding P3 than those that were later missed. In this line, the decreased P3 amplitude in ADHD would point to a deficient WM encoding process. This way of interpreting P3 amplitude falls within the frame of the “context updating theory” proposed by Donchin and Coles^32^ which suggested that P3 amplitude reflects the effort to continuously update new relevant information to the representation held in WM. Another view (non-necessarily opposite) suggests that P3 amplitude reflects the allocation of attentional resources necessary to categorize stimuli for encoding and to discriminate its relevance in the retrieval stage^64^. Although the exact meaning of WM-related P3 amplitude modulations is still a matter of discussion, the correlation between its amplitude and WM efficiency seems reliable. WM representations are flexible and can be modulated dynamically according to changing goals and expectations^68^, and such process requires dynamic allocation of attention and representation updating which modulates P3 amplitude.

Regarding the retrieval stage, we found larger P3 amplitude for the “Same” condition compared to the “Different” one. These effects are in line with previous results described as the new-old effect in studies of recognition memory^69^, where larger P3 amplitudes are reported for the old items compared to new ones. It has been suggested that such amplitude modulation reflects activity from a recollection-sensitive regions in the lateral parietal cortex, functionally indexing the representation of recollected information^66^. Alternatively, in the context of a change detection task, this amplitude modulation could also be interpreted as reflecting a more exhaustive memory search in the “Same” condition until the presence of a change has been ruled out. The latter view is consistent with the notion that correctly detecting change implies recollection while detecting “sameness” or absence of change, involve identifying familiarity^70^. Taken together the results from the P3 component presented here suggest that both the encoding and retrieval WM processes could be compromised in ADHD.

The amplitude of Encoding and Retrieval P3 components were significantly correlated both in the same and in the different trial types. This correlation was apparently driven by the results from the non-ADHD group. Nevertheless, a follow up analysis comparing correlations coefficients between groups resulted non-discriminative, probably due to limited sample size. Given the sequential nature of the task and the probable participation of attention both during encoding and retrieval the correlations of this neurophysiological indicators was an expected result but needs further confirmation using a larger sample of participants.

Another WM process that could potentially account for the impairments seen in ADHD is the binding process itself. That is the construction of an integrative process of objects in WM. In the current work we did not find significant difference between groups regarding the binding condition (Shape-Only Vs. Color-Shape), neither behaviorally nor electrophysiologically. Even though, the Color-Shape condition resulted in larger reaction times in all participants. To our knowledge this is first study to assess WM binding processes in ADHD. The lack of group differences could be explained by that fact that the neural system subserving binding processes has been reported to involve a posterior network of parietal, temporal and occipital areas^20^, and not the Prefrontal Cortex anterior executive network usually reported to be affected in ADHD^71^. It should be noted that parietal regions have also been reported to be affected in ADHD^72^. Nevertheless, metanalytic studies suggest that ADHD impairments in different neural networks are closely related to the tasks or domains being evaluated^71,73^. While parietal dysfunctions in ADHD have been mostly related to attentional orienting tasks (right inferior parietal cortex)^73^, visuospatial working memory tasks have been mostly correlated with frontal regions dysfunctions^74^. These findings have important implications for current understanding of the functional organization of WM which we address in the next section. According to our results, binding functions carried out in WM seem to be intact in ADHD. Nevertheless, more specific studies are undoubtfully needed to explore in depth WM binding in this condition^71^.

In this context, our results could be interpreted as a failure in ADHD adolescents to update WM representations to accomplish task demands or a failure in the prioritization of representations as suggested by Myers *et al*.^75^. This could potentially impact different subprocesses. First, during the encoding stage reduced P3 amplitude could reflect deficient attention allocation to the relevant aspects when creating a representation. Then, during the retrieval stage the smaller P3 amplitude in ADHD, which also fails to discriminate between “Same” and “Different” (as it occurs in the non-ADHD group) could be interpreted as evidence of a widespread failure to assign post-selection priority to the representations held in memory and their posterior update to solve the task at hand. This seems to be especially clear when the test stimuli were different from the study ones.

### Implications for the functional organization WM

This study provides valuable evidence to further assess recent positions regarding the functional organization of WM. A question that has received substantial attention in recent years is whether binding functions operating in WM require additional attentional resources^17–19^. Thoroughly conducted experiments have manipulated attention during visual WM binding tasks using different approaches and all have failed to demonstrate that binding requires resources above and beyond those needed to process individual features. Baddeley^12^ envisaged that the episodic buffer was the WM component where such binding operations would occur supported by attention. Should this proposal be valid, any WM operation requiring binding would be dramatically affected if attentional resources are not available during such operation. Clearly, that was not the case in the series of experiments above described. These consistent findings led Baddeley and collaborators^16^ to revise the WM models and reconsider the function of the episodic buffer. The new revision suggests that low-level binding functions, such as those needed to integrate surface features and form objects identity, can be carried out outside the episodic buffer, being areas in the posterior part of the brain likely neural correlates^20^. However, a potential limitation of these experiments is that attention was experimentally manipulated making it possible that individual differences in attentional resources would have impacted on such outcomes. A more reliable approach would involve individuals with attentional impairments such as those diagnosed with ADHD. In the current study we addressed this issue in such a population. We have confirmed that individuals with attention impairment, as demonstrated by their clinical profiles and general WM functions, are still able to hold bound information in WM. This is the first study reporting such findings which, to the authors’ views, support the notion that such binding operations could be automatic.

A potential account for such relation between attention and binding function of WM has been linked to the type of attention needed to support this function. While executive attention seemingly driven by functions of the prefrontal cortex might be crucial for binding operations happening within the episodic buffer^16^, other bottom-up low-level attentional functions might support the binding of surface features within integrated objects. Such functions, which are seemingly supported by a posterior network involving parietal, temporal and occipital areas^20,45,49,76^, might be less vulnerable to conditions impacting on attention such as ADHD or even depression^40^.

Recent studies have pointed to a functional integration deficit of connectivity-based pathophysiologic process in ADHD^77–80^. Control networks recruited during WM tasks are sensitive to neurodevelopmental factors which affect the patterns of connectivity integration/and segregation^81^. A significant body of literature suggests that frontal networks, as those sub-serving WM, seem to be affected in ADHD^82–85^. This would explain the overall WM impairment seen in these patients in the current study.

### Limitations and future directions

The current study has limitations that should be addressed in future studies. First, current and previous studies use no nameable geometric shapes and non-primary colors trying to avoid phonological coding of non-verbal material^20,45^. This approach reduces but can’t ensure the complete avoidance of implicit verbal rehearsal^86^. The presence of such strategy was not measure and its potential impact can’t be ruled out. Future studies should consider this in their design to directly address this issue. Second, ADHD is a complex, multisystem and highly heterogenic condition. Although inattention is probably its most consistent characteristic, the presence of the diagnosis cannot be equated to a constant or stable deficit. There is large intra and inter subject’s variability. The inferences regarding the relations between WM, binding and Attention should be further investigated using different experimental designs and larger sample sizes. Third, future studies should separate more systematically attention and working memory and also should address the potential impact of stimulant medication on WM deficits in ADHD, as well as, different ADHD subtypes.

This work opens a new agenda investigating the role of inter-coupling among attention and WM process and networks in ADHD and other neuropsychiatric conditions that impact on these cognitive abilities. In sum, studying the interaction between attentional guided dynamic prioritization and WM in ADHD could be a promising approach to understand the pathophysiology of the condition and to refine understanding of models of memory.

## Data Availability

The datasets generated during the current study are available from the corresponding author on reasonable request.
